# Use of a Humanoid Robot in Supporting Dementia Care: A Qualitative Analysis

**DOI:** 10.1177/23779608231179528

**Published:** 2023-06-04

**Authors:** Yo-Jen Liao, Ying-Ling Jao, Marie Boltz, Olayemi Timothy Adekeye, Diane Berish, Fengpei Yuan, Xiaopeng Zhao

**Affiliations:** 1 8082 Pennsylvania State University, Ross and Carol Nese College of Nursing, University Park, PA, USA; 2 Department of Mechanical, Aerospace, and Biomedical Engineering, University of Tennessee, Knoxville, TN, USA

**Keywords:** dementia, Alzheimer's, humanoid social robot, perception, pepper

## Abstract

**Introduction:**

Cognitive impairment significantly affects independence in persons with dementia, and consistent supervision is often needed. While interest has arisen in using humanoid robots, such as Pepper, to assist with daily caregiving activities, little is known about the perceptions of using Pepper to assist people with dementia.

**Objective:**

This study aimed to explore the perceptions of nonhealthcare workers, care partners, and healthcare workers on the use of a Pepper robot in dementia care.

**Methods:**

This was a secondary qualitative analysis. Data were collected from a pilot study conducted from November 2020 to March 2021 using an online survey. The survey consisted of quantitative and qualitative questions; this study only focused on the qualitative responses. The detailed procedures and the quantitative results were published elsewhere. Participants included nonhealthcare workers, care partners, and healthcare workers.

**Results:**

A total of 194 participants responded to the open-ended question. Participants described potential benefits of Pepper including assisting with daily activities, monitoring safety and medication use, initiating reminders, and promoting activities and social interactions. Participants had concerns about privacy, cost, poor acceptance/trust, Pepper making mistakes, limitations in environmental navigation and responding to emergencies, misuse of Pepper, and Pepper replacing humans. Participants suggested that Pepper should be tailored to each individual's background, preferences, and functions and recommended improving the logistics of using Pepper, offering more emotional support and responses, and using a more natural appearance and voice.

**Conclusion:**

Pepper may support dementia care; yet some concerns need to be addressed. Future research should consider incorporating these comments when designing robots for dementia care.

## Introduction

Worldwide, more than 55 million people are currently diagnosed with dementia, and that number is estimated to grow to 78 million by 2030 (World Health Organization [WHO], 2021). Cognitive decline profoundly impacts the health and daily functioning of both the person with dementia as well as their care partner. Impairments in memory, communication ability, and performance of activities of daily living (ADLs) require a significant amount of support and assistance from those providing care (Gale et al., 2018; Giebel et al., 2014; Mograbi et al., 2018; van Wyk et al., 2017). As the disease progresses, people with dementia need a higher level of assistance with household tasks and daily personal activities, consistent supervision (Wang et al., 2014), and psychosocial support (van Wyk et al., 2017).

Many non-pharmacological approaches and strategies can improve the lives of people living with dementia and their care partners. The goals of these interventions are optimizing cognitive and physical function, safety, improving a sense of well-being, decreasing behavioral expressions of distress, and avoiding unnecessary psychoactive medication in the person living with dementia (Ballard et al., 2016; Brodaty & Arasaratnam, 2012; de Oliveira et al., 2015; Neville et al., 2014). Non-pharmacological treatments that focus on physical, emotional, and mental activity not only improve the health and quality of life (QOL) of the person living with dementia, but they also contribute to the well-being of care partners and support a positive relationship between the care partner and the care receiver (Burns et al., 2003; de Oliveira et al., 2015; Gallagher-Thompson et al., 2003). Although more research is warranted, new technologies such as socially assistive robots (SARs) have emerged to support a wide range of therapeutic approaches for people living with dementia, such as companion, recreational therapy, and sensory therapy (Abbott et al., 2019; Bemelmans et al., 2012; Chu et al. 2017; Jøranson et al., 2016; Kang et al., 2020; Leng et al., 2019; Moyle et al., 2017).

## Review of Literature

**Figure 1.**
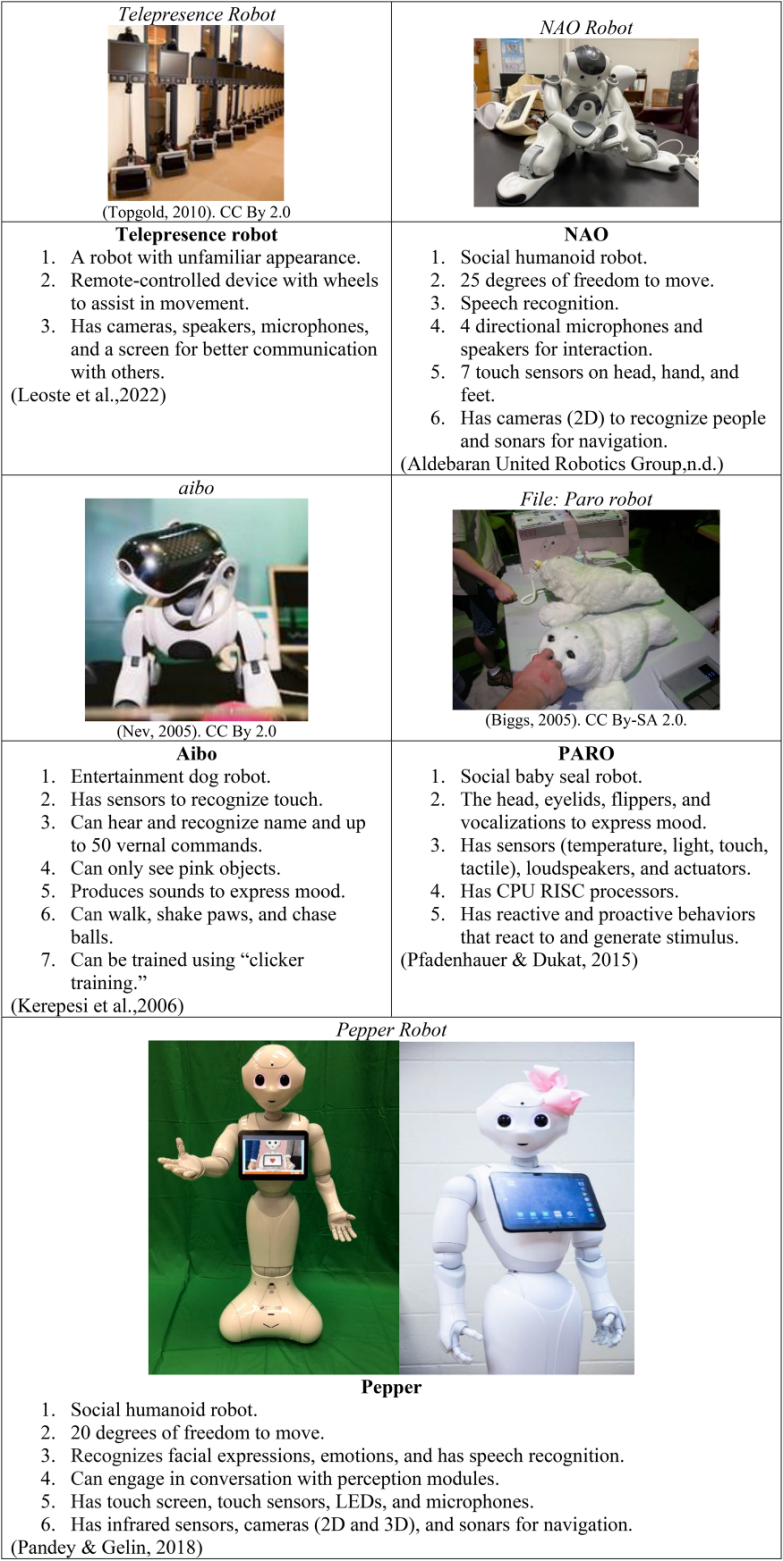
Robot Descriptions.

SARs generally refer to robots that can provide assistance and interact with users (Bemelmans et al., 2012). Robots come in various sizes and appearances and different functions are provided (Zuschnegg et al., 2021). SARs utilize interaction strategies including speech, facial expressions, and communicative gestures to initiate reminders, motivate physical activity, promote safety, prompt social engagement and leisure activities, and provide companionship and entertainment (Góngora Alonso et al., 2019; Huschilt & Clune, 2012; Valentí Soler et al., 2015). SARs, including pet-like, humanoid robots, and robots with unfamiliar appearances, have been investigated or explored in dementia care (Figure 1) (Casey et al., 2020; Chu et al., 2017; Jøranson et al., 2016; Kang et al., 2020; Koh et al., 2021; Moyle et al., 2020; Wang et al., 2017, Yuan et al., 2021a). Some evidence has shown that SARs are beneficial for people with dementia. Specifically, pet-like robots are designed to look like animals. They are beneficial in promoting feelings of companionship, motivating activities, and improving affect, social interactions, and QOL. They are also associated with reduced behavioral symptoms of distress in people with dementia (Jøranson et al., 2016; Kang et al., 2020). Robots with unfamiliar appearances, such as Pudu robots (telepresence robot) with wheels to move around and a tablet at the top to help with communication and interaction with other people, were employed to provide mental and emotional health care to patients in isolation (Ruiz-del-Solar et al., 2021). Other SARs with unfamiliar appearances (Giraff and VGo) were reported to be useful in enhancing the connections and interactions between people with dementia and their families (Moyle et al., 2020). Non-humanoid robots have limited social-engaging capabilities due to their structure and design (Huschilt & Clune, 2012). Recent research has begun to explore the use of humanoid robots to expand potential functionality in caregiving support for persons with cognitive and/or functional impairments (Sato et al., 2020; Zuschnegg et al., 2021). It is estimated that the cost of humanoid robots is approximately $10,000 for the NAO robot and from $22,000 to $35,000 for the Pepper robot; whereas pet robots cost approximately $3,000 (Aibo companion robot) or $6000 (Paro) (Aldebaran United Robotics Group, n.d.; Fracasso et al., 2022) (Figure 1).

Humanoid robots have the features of a human body with a face and arms and mimic human behaviors (Prakash & Rogers, 2015). These human-like characteristics and detailed facial expressions can facilitate interactions with people (Prakash & Rogers, 2015). Humanoid robots are multi-function robots that can move around with users and assist with mobility and tasks (Prakash & Rogers, 2015). These robots are useful in assisting with multiple healthcare tasks and daily chores such as updating calendars, providing medication reminders, facilitating cognitive training, walking with users, and rehabilitation (Andtfolk et al., 2022; Ismail, 2021). Moreover, using SARs during the COVID-19 pandemic could help healthcare workers complete caregiving tasks while minimizing infection transmission (Ismail, 2021). Pepper is one type of humanoid robot that can recognize emotions through facial expressions and voices and interact with users verbally and nonverbally (gestures, eye contact, and other body language) (Ishiguro et al., 2016). Pepper is manufactured by the Aldebaran United Robotics Group and is 120 centimeters tall, has a touch-screen tablet in the front of the chest, wheels to move around, and arms and fingers (Pandey & Gelin, 2018) (Figure 1). It has natural facial expressions, touch sensors, 3D cameras and sonars for navigation, and speech recognition up to 22 languages with additional software installed, including English, Arabic, Mandarin, Taiwanese, Czech, Danish, Dutch, Finnish, French, German, Greek, Italian, Japanese, Korean, Norwegian, Polish, Brazilian, Portuguese, Russian, Spanish, Swedish, and Turkish (Pandey & Gelin, 2018; RobotLAB, 2020).

Pepper has been used in supporting caregiving tasks. During the pandemic, Pepper has been used in hospitals to assist in implementing COVID-19-related measures (e.g., masking and social distancing) in the Czech Republic and Germany (Musa, 2020; Tyagi, 2021). For example, Pepper can help monitor body temperature and remind people to disinfect their hands and wear masks in hospitals and shopping malls (Musa, 2020; Tyagi, 2021). Pepper has also been used to help reduce stress and anxiety among children who are hospitalized (Adam, 2018). Additionally, research evidence supports the use of Pepper in clinical care. One study utilized Pepper to assist with an exercise program for hospitalized patients with mental illness and impaired physical function and showed that participants had better engagement and greater interactions with nurses (Ujike et al., 2019). In addition, a qualitative study compared participants’ perceptions of interacting with the tablet on Pepper only and with the entire Pepper in performing cognitive-motor activities (Feingold-Polak et al., 2018). Results revealed that among 20 participants (10 younger and 10 older healthy adults), 70% preferred interacting with Pepper over using its tablet alone because interacting with Pepper was more interesting and human-like (Feingold-Polak et al., 2018).

Despite the potential of humanoid robots to engage users and support care, Pepper has not specifically been implanted in assisting with care for persons with dementia in clinical practice. In addition, limited research has been conducted on Pepper, specifically in people with dementia (Sato et al., 2020). People with dementia and their care partners may have special needs different from healthy adults, people with mental illness, and persons with impaired physical function. Understanding users’ perceptions of robots would facilitate the successful implementation of robot interventions. This is especially important for the vulnerable older adult population. Sato et al. (2020) conducted a qualitative case study using Pepper in older adults with schizophrenia and/or dementia and showed that using humanoid robots in long-term care still needs improvements in technology to improve communication and promote activities in users (Sato et al., 2020). Another study (Zuschnegg et al., 2021) focused on the expectations of 52 dementia care partners and healthcare workers on using humanoid robots through a qualitative study. Zuschnegg et al. (2021) showed the participants a video of Pepper as an example and explored participants’ perceptions of humanoid robots in general. Results revealed that the participants mostly had positive expectations of humanoid robots in providing support for avoiding danger, improving communication, assisting with daily activities, and providing recreational activities (Zuschnegg et al., 2021). However, negative expectations were also mentioned including the potential risks of decreased human interaction and concerns about the robot's responsiveness during emergencies (Zuschnegg et al., 2021). While the study from Zuschnegg et al. (2021) examined the use of humanoid robots, it only focused on care partners and healthcare workers and did not explore the perspectives from nonhealthcare workers. The authors explored perceptions of non-healthcare workers, care partners, and healthcare workers on the use of Pepper in supporting dementia care. Furthermore, the study from Zuschnegg et al. (2021) showed the functions of Pepper as an example of humanoid robots, but they explored the perspectives of the participants on humanoid robots in general and not specific to Pepper. This study focused on the opinions of the participants on Pepper specifically. To address this gap, the purpose of this study was to explore the perspectives of nonhealthcare workers, care partners, and healthcare workers in using Pepper in supporting care for individuals with dementia.

## Methods

### Research Question

What are the perceptions of nonhealthcare workers, care partners, and healthcare workers on the use of Pepper in supporting care for people with dementia?

### Study Design and Samples

This qualitative descriptive study explores perceptions from three groups of participants (nonhealthcare workers, care partners, and healthcare workers) in using Pepper to support care for persons with dementia. This study was performed online by distributing a survey via social media including Facebook, LinkedIn, WeChat, and Twitter, primarily in the United States (US).

### Inclusion and Exclusion Criteria

Inclusion criteria were individuals aged 18 years or older. There were no exclusion criteria.

### Procedures and Data Collection

An online survey was developed via the web survey design tool QuestionPro that contained open- and close-ended questions. The details of the research procedures were published elsewhere (Yuan et al., 2021b). In this study, data from the one open-ended question was analyzed. The survey was based on the Almere model (Heerink et al., 2010), a scale designed to measure older adults’ acceptance of SARs (Cavallo et al., 2018; Sancarlo et al., 2016). Participants filled out informed consent online and watched a 3-min introduction video about Pepper and how Pepper may be used to support care for people with dementia. The functions of Pepper presented in the video included reminders to take medicine, provide entertainment, walk with users, motivate exercise, help calling friends and families, and interact with persons with dementia (Yuan et al., 2020). Subsequently, they were asked to fill out the survey which included four questions related to demographics and 32 Likert-scale questions developed in this study about the acceptability of the robot's appearance, functions, and usage.

Participants were categorized into care partners (including family and friends providing care for people with dementia), healthcare workers (including healthcare personnel and healthcare professionals), or nonhealthcare workers based on self-report. Care partners were defined as people who reported they took care of people with dementia informally, such as taking care of their relatives and friends with dementia. Healthcare workers were defined as paid care partners who reported they worked in dementia-related facilities/units, such as long-term care, hospital, or home care. Nonhealthcare workers were those who reported they did not provide care for people with dementia (formally or informally). One open-ended question was included at the end of the survey that asked “do you have any additional comments, suggestions, or concerns you would like to share in order to develop a robot to assist in providing Alzheimer's care?” No compensation was provided for completing this survey. Data collection took place from November 12, 2020, to June 25, 2021.

### Ethical Considerations

The Institutional Review Board (IRB) of the University of Tennessee, Knoxville, granted approval for this study. Protocols (protocol NO. UTK IRB-20-06032-XM) were followed, including participant anonymity, privacy maintenance, and informed consent before starting the survey.

### Data Analysis

A qualitative content analysis was performed using Excel. First, the responses of the open-ended question were exported and organized in an Excel file for data analysis. Second, three researchers (Y-JL, Y-LJ, and OTA) read the data and discussed coding strategies with the research team. Third, the three researchers (Y-JL, Y-LJ, and OTA) independently coded the data in an Excel file and discussed initial codes with the research team. Fourth, the codes were compared for similarities and discrepancies. Similar codes were grouped together, and disagreements were reconciled with a fourth researcher with extensive experience in qualitative research (MB). Fifth, researchers (Y-JL, Y-LJ, and OTA) summarized similar codes and categorized the codes using Excel into eight categories (design, functionality, logistics, cost, trust, acceptance, privacy, and concerns about replacing human) and interpreted the results. The data from nonhealthcare workers, care partners, and healthcare workers were analyzed separately to distinguish the comments from different perspectives. Rigor was supported through the use of multiple coders and review findings with the research team.

## Results

### Description of Participants

A total of 194 participants responded to the open-ended question within the survey. These participants were nonhealthcare workers (*n*  =  89, 45.9%), care partners (*n*  =  94, 48.5%), and healthcare workers (*n*  =  11, 5.7%). In the care partner group (*n*  =  94), seven of them also worked in healthcare (six nurses and one physician). In the healthcare worker group (n  =  11), their healthcare professions were nurses (n  =  7), therapists (n  =  2), and paid in-home caregivers (n  =  2). Most of the participants were females (n  =  134, 69.1%) from the US (n  =  189, 97.4%). Age-wise, slightly over half of the participants (n  =  102, 52.6%) were 65 years or older and had a postgraduate degree (n  =  99, 51%) (Table 1).

**Table 1.** Demographics.

	Nonhealthcare workers (*N* = 89)	Informal care partners (*N* = 94)	Healthcare workers (*N* = 11)	Total (*N* = 194)	
Gender					
Female	51 (57.3%)	73 (77.7%)	10 (90.9%)	134	(69.1%)
Male	37 (41.5%)	20 (21.3%)	1 (9.1%)	58	(30.0%)
Age					
Under 50	23 (25.8%)	13 (13.8%)	4 (36.4%)	40	(21.6%)
50–65	16 (18.0%)	34 (36.2%)	2 (18.2%)	52	(26.8%)
66–75	30 (33.7%)	34 (36.2%)	4 (36.4%)	68	(35.1%)
76–85	18 (20.2%)	12 (12.8%)	1 (9.1%)	31	(16.0%)
Above 86	2 (2.2%)	1 (1.1%)	0 (0.0%)	3	(1.5%)
Country					
US	87 (97.8%)	91 (10.1%)	11 (100.0%)	189	(97.4%)
Canada	1 (1.1%)	0 (0.0%)	0 (0.0%)	1	(0.5%)
Taiwan	1 (1.1%)	0 (0.0%)	0 (0.0%)	1	(0.5%)
India	0 (0.0%)	1 (1.1%)	0 (0.0%)	1	(0.5%)
Germany	0 (0.0%)	1 (1.1%)	0 (0.0%)	1	(0.5%)
Europe	0 (0.0%)	1 (1.1%)	0 (0.0%)	1	(0.5%)
Educational level					
High school graduate	5 (5.6%)	1 (1.1%)	1 (9.1%)	7	(3.6%)
Some college	13 (14.6%)	21 (22.3%)	0 (0.0%)	34	(17.5%)
College graduate	28 (31.5%)	24 (25.5%)	2 (18.2%)	54	(27.8%)
Postgraduate	43 (48.3%)	48 (51.1%)	8 (72.7%)	99	(51.0%)
Occupation	Not reported				
Nurse		6 (6.3%)	7 (63.6%)	13 (6.7%)	
Therapist		0 (0.0%)	2 (18.1%)	2 (1.0%)	
Physician		1 (1.1%)	0 (0.0%)	2 (1.0%)	
Paid in-home caregiver		0 (0.0%)	2 (18.1%)	2 (1.0%)	

### Findings

Participants’ responses on the use of Pepper to assist with care for individuals with dementia were categorized into eight main areas: design, functionality, logistics, cost, trust, acceptance, privacy, and concerns about replacing humans. The categories and subcategories of responses and example quotes are presented in Table 2.

**Table 2.** Category, Codes, and Quotes.

Categories	Codes	Quotes
**Design**	Positive views of robots	“*Very intriguing, possibly life-changing creation.”* (Nonhealthcare worker).
	Negative perceptions of robot appearance	“*Looks like a toy to support caregiving I build Robots some in my spare time and Feel like this Robot looks too much like a toy to really be considered as a serious replacement for a caregiver.*” (Nonhealthcare worker).
	Positive perceptions of robot appearance	“*Color on robot – not sterile. Great idea. Good luck.*” (Nonhealthcare worker).
	Suggestions for improving robot appearance	“*The ‘robots’ could be more ethnically diverse…in color, speech and perhaps come ‘pre-loaded’ with historical information germane to the patient*.” (Nonhealthcare worker).
		“*The robot is a machine and does not need to look like a human or behave like a human. It has some beneficial uses, however considering it as a companion/caregiver is a little unnerving*.” (Nonhealthcare worker).
	Voice needs improvement	“*I don’t know why you would choose a mechanical sounding voice when Google Mini and other inexpensive devices have near human sounding voice.”* (Nonhealthcare worker).
		“*The machine/computer voice would be extremely startling to a patient*” (Informal caregiver).
	Suggestions for voice/speech improvement	“*I think it is imperative that voice quality be normal and readily perceived and tailored to each person's hearing and language skills.”* (Formal caregiver).
		“*I would be mindful of words that are used by the robot. We like to call the shower room at care facilities the “spa.” However, most geriatric people in facilities have never been to a spa. We fail to realize that many of them didn’t even have showers, only bathtubs. I wouldn’t use new words that an elder wouldn't understand their meaning*.” (Informal caregiver).
	Tone and use of words need improvement	“*The bot (robot) is programmed to assume that the subscriber is neglecting to take their prescribed medicine and goes into a mode of ‘shaming them to comply. Only a human should do this unless you want the robot severely damaged*.” (Nonhealthcare worker).
**Functionality**	Support and suggestions for robot function in dementia care	“*Human services should be done by humans. Assign robots impersonal tasks…* *cleaning, calculating, record keeping, etc.*” (Informal caregiver).
		“*Assess the person served by a robot for hearing, vision, mobility and program robot to meet needs. Assess social likes/dislikes, for brain/cognitive exercises. Assess what makes the person happy, program robot to trigger those responses. Program robot with components of person's jobs or hobby, i.e., military, woodworker, artist, teacher etc*.” (Informal caregiver). “*Many functions could be integrated into apps that do not require a robot to be physically in*
		*the room, loaded into a car, or out navigating lawns, sidewalks, and curbs. Small devices in each room (think Amazon Echo) with cameras and wearable AI (Artificial Intelligence) make much more sense to me*.” (Nonhealthcare worker).
	Benefits of robot to dementia care	“*Both passed away, but my husband had LB dementia and my mother ALZ. This might have given me peace of mind a little longer in leaving them alone at home for short times.*” (Informal caregiver).
		“*I think a robot that could assist the client as well as the caregiver is an excellent way to keep the client in his or her own home environment*.” (Informal caregiver).
	Benefits of robot to people without dementia	*“I think this is a great idea for Alzheimer's care. I can also see this being used for patients that have had strokes. My sister is recovering from the effects of COVID after having a Stroke, being on a Ventilator and a Tracheostomy. She is having rehabilitation. She is coming home soon. She is having to learn to walk again and has no use to her left arm/hand and limited use to her left hand and arm. I feel COVID patients with these disabilities could also have the need for the Robot. She will require home health. Robots are great for people Mentally and Physically…* *:).”* (Informal caregiver),
	Concerns about robot use	*“If it were to develop to the point that a robot was brought in and basically tells me what to do and when to do it, where does my personal thinking functions go?*” (Nonhealthcare worker).
	Concerns about robot in dementia care	“*Many of my answers depend on additional factors. For instance, this particular robot may not be ideal for physical safety (e.g., may not respond quickly enough to prevent a fall). It might, on the other hand, recognize motor behaviors and predict the likelihood of fall – in this instance, it could ask the individual to sit down.*” (Nonhealthcare worker).
		“*I sincerely doubt a robot would be able to understand or converse with an Alzheimer's patient. Such patients often use the wrong words for things, places, and in an inconsistent manner. They use the wrong verbs and refer to people by incorrect names. The robot needs to be able to understand sentences such as “can we go to* *….* *you know* *….* *last week (meaning yesterday), where we saw Greg (meaning Philip) … the place where those colored things that smell god are (meaning the bakery where we say her neighbor with the beautiful garden*.” (Informal caregiver).
	Too ambitious to develop an empathetic and all-knowing robot	*“All of my answers could have been “don’t know,” simply because I haven’t “been there.” But I tried to answer as though I were the one receiving the robotic care. An empathetic, all-knowing robot is quite an ambitious undertaking. Nothing is mentioned re cost of such a robot, but I Imagine any caregiver would be grateful for any relief from the mental strain of caring for such a patient. Good luck in your journey!”* (Nonhealthcare worker).
	Suggestions for implementation	“*Different people will have different needs. It will be important for no relevant things to be dumped so the options are relevant and easy to sort through (versus having interfaces cluttered with functions someone won’t use, making it hard to find the few things that do matter)*.” (Nonhealthcare worker).
		“*Having been a caregiver, different stages require vastly different amounts of time, care, and input. I.e., the robot would not be useful to give map directions to someone who has progressed to the point that they cannot make sense of directions, or void recognition would not be useful if the patient cannot create understandable/logical sentences.*” (Informal caregiver).
**Logistics**	Devices to remotely control robot	*“The robotic industry has come a long way over the last ten years. Robotics is being taught to high schoolers and I believe within 10 years many jobs will be lost to advancement of such technologies. However, this is our future and it will happen eventually like evolution. An elderly person could also have a smart watch or similar device to be able to remotely control it as well.”* (Nonhealthcare worker).
	Technical support	*“Real time technical support would be extremely important and should be easily accessible.”* (Informal caregiver).
	Environmental navigation and limitations	“*Mobility issues of the robot, example, a couple stairs to go outside the house for a walk*.” (Formal caregiver).
		“*The robot shown does not appear to have the ability to go up or down a single stair. If so, it would have limited ability to assist in many homes or other living arrangements and outdoor locations*.” (Formal caregiver).
	People with dementia using robot	“*I think it is a good idea. However, from my experience caring for my mother with Alzheimer's, I can’t imagine an Alzheimer's patient having anywhere near the cognitive or emotional ability to LEARN to use any of the functions, if learning such thing is necessary*.” (Informal caregiver).
		“*I have a family member who has progressive Alzheimer's. Some days she is unable to remember who we are. If she has trouble distinguishing who we are, with significant more detailed faces, bodies, and personalities, than the robot depicted, then I am not sure how well she would be able to recognize/work with a face/body she doesn’t remember*.” (Nonhealthcare worker).
**Cost**	Cost and affordability	“*My concern however is more of cost of use. People that can afford this type of technology are likely able to afford personal care, under-cared for sector is the lower income sector. Those with little to no family. I believe they would benefit the most from this type of technology, but is that realistic?*” (Informal caregiver).
**Trust, acceptance, and privacy**	Information privacy	*“Patient privacy (cyber-security) could be an issue if the robot holds too much information on that patient. You guys need to add anti-theft measures (like self-destruction).”* (Nonhealthcare worker).
	Concerns about trust and acceptance of the robot	“*As a nurse who has cared for those with Alzheimer's in the hospital, my first thought regarding robots would be that they could create a sense of fear in those with Alzheimer's. I feel like they are often fearful at those trying to help them (some of whom are family members) that a robot might really scare them*.” (Formal caregiver).
		“*Acceptance of a robot in an Alzheimer's life will strongly depend on the patient's background and their present emotional and cognitive state. They may see the robot as a friend or an enemy depending on their cognitive state*.” (Informal caregiver).
**Concerns about robot replacing human**	Robot cannot replace human	“*This is against my religious beliefs to replace humans with non-human robots, and I am totally against this type of technology. It is GODLESS and USELESS*” (Nonhealthcare worker).
		“*My concern is that a robot with the artificial intelligence expressed in the video and in your questions will replace human caregivers and the contact and emotional support they provide. I am afraid too many families might place a relative with AD or dementia under the long-term care of a robot. I can’t imagine being under the full-time care of a walking, talking robot.*” (Nonhealthcare worker).

#### Robot design

Participants provided their perspectives on the design of Pepper, specifically the Pepper's appearance and verbal communication, and offered suggestions for improvement. Nonhealthcare workers and care partners had both positive and negative perceptions of Pepper's appearance for supporting care for persons with dementia. For positive perceptions on Pepper's appearance, one participant commented that “the color of the robot is not sterile, which is good.” On the other hand, around 30 participants in these two groups commented that the robot looked “frightening and creepy,” its gestures were “unnerving,” and that people with dementia might fear Pepper. Some participants also commented that Pepper looked like a “toy” or a “child,” thus, it would not be taken seriously. Healthcare workers provided suggestions on improving the appearance of Pepper but did not express the specific positive or negative perception of Pepper's appearance.

Some participants across the three groups provided suggestions on improving Pepper's appearance. For example, they suggested that Pepper be taller, stronger, have more realistic eyes, have two legs, have more color, and be personalized and ethnically diverse. Interestingly, participants in the nonhealthcare worker and care partner groups had different views on whether Pepper should look more or less human-like. Some participants from the nonhealthcare workers and care partners suggested that Pepper look more human-like; others (nonhealthcare workers) suggested the opposite. Furthermore, one participant mentioned that Pepper should be designed with fewer points of failure, such as replacing joints with wheels to lower the risk of the Pepper breaking.

Participants across the three groups criticized the voice of Pepper, with comments that the voice was “too artificial,” “pixie,” “mechanical,” “annoying,” and “off-putting.” Moreover, participants commented that Pepper's voice was difficult to hear and understand, even for people without cognitive impairment. Suggestions for improvement included making the voice more soothing, natural, clearer, and adult-like with variance in tones. Furthermore, participants commented that adding lip movements, changeable pitch and volume, and slower speech pace were essential functions to consider for people with hearing impairments.

Other suggestions were that the language, voice options and patterns, and voice quality should be tailored to individuals’ preferences, background (occupation, culture, and socioeconomic status [SES]), education levels, and cognitive and functional levels. Several participants in the nonhealthcare worker group also suggested that Pepper should avoid “talking down” to the person with dementia and assuming that the person with dementia will not be adherent with their plan of care.

#### Functionality

Participants across the three groups voiced support for Pepper's function in assisting with dementia care and easing care partner stress. Specifically, participants commented that Pepper could help with the following:

1. Providing emotional support, social connection, and companionship: offering emotional responses and support can calm anxiety and fear. Pepper can also provide companionship and assist with social interaction and connection through telephone or video conference.
2. Prompting activity engagement with sensory stimulation and leisure activities: provide activities (e.g., games, music, scents, pictures, meditation) and stimulate physical activities (e.g., yoga and exercise).
3. Facilitating communication: use word reminders or pictures when people with dementia are experiencing communication challenges.
4. Helping communication among people with hearing impairments: add subtitles, lip movements, and language recognition to facilitate communication.
5. Monitoring and alerting care partners or healthcare workers during unsafe situations: monitor the condition of people with dementia (e.g., vital signs, safety, medication use) and prevent risky situations and injuries (e.g., falls) were suggested as essential functions. Alert care partners or healthcare workers when people with dementia are in unsafe situations (e.g., abuse) and take the user to shelter during emergencies.
6. Assisting with caregiving tasks and promoting independence in persons with dementia: provide physical assistance, set reminders (appointments, medication use, hydration, and toileting), educate care partners, support caregiving, and assist with ADLs (e.g., dressing, mobility, toileting, and eating).
7. Individualization: assess dependency and tailor Pepper's functions to individual preferences and needs according to user background and cognitive and functional level.

Participants suggested starting with Pepper's core functions but did not specify what core function to start with. Participants were uncertain about what level of cognitive impairment would benefit from Pepper. Some participants also commented that Pepper might be helpful for people without dementia who have functional impairments, people without families, or individuals that lack human interactions. Notably, several participants in the nonhealthcare worker and care partners groups pointed out that other existing devices or applications can replace some of the Pepper's functions with lower prices, such as using Alexa or Amazon Echo for home monitoring or Siri for setting reminders.

Some participants raised concerns about the need to supervise Pepper to prevent mistakes. One participant mentioned that Pepper should be assigned to do impersonal tasks, such as cleaning the house or assisting with groceries, rather than human services. Furthermore, Pepper's capacity to protect people with dementia from dangerous situations was also mentioned, and there were concerns that Pepper may not be able to respond to people with dementia quickly enough if they had a fall. Finally, concerns about misuse of Pepper were also mentioned, for example, utilizing them to replace staff in long-term care settings.

The nonhealthcare worker group raised some concerns that people, such as people with dementia and their care partners, may overly rely on Pepper and stop thinking. Additionally, some expressed doubts about Pepper's capabilities, “It seems to be too ambitious to develop an all-knowing robot,” for example. Participants also questioned Pepper's function in monitoring and reminding (medication and bathroom use), strength in physically supporting heavy patients, communication effectiveness, and capacity to assist in daily activities (robot causing falls and accidents and not being able to detect pills, open bottles, handle toileting, and have conversations with people with dementia).

When implementing Pepper in dementia care, most participants in the nonhealthcare workers and care partners groups proposed that projects start simply with essential functions but did not specify what the essential functions should be. Pepper should be tested in older adults, in general, to address issues prior to using it in people with dementia. Also, one participant suggested that Pepper should be used in a controlled environment such as group homes. Other suggestions included approaching people with dementia with usual care rather than monitoring them like a spy, assisting but not directing activities, providing individualization (naming Pepper as someone meaningful), and assessing patient changes and adjusting care accordingly. One participant commented that Pepper's emotions should be more fully developed. Finally, some care partners were concerned about using Pepper with people with dementia while they are alone and suggested that primary care partners should be around for supervision.

#### Logistics

One participant in the nonhealthcare workers group suggested that devices, such as smartwatches, should control Pepper remotely. Another informal care partner mentioned that real-time technical support with easy accessibility should be provided for troubleshooting. Several participants raised concerns about the Pepper's battery function and adding battery/recharge and backup functions were suggested. Moreover, several environmental navigation limitations were noted by participants across the three groups. They were primarily concerned about space in the home, mobility on uneven floors and stairs, Pepper’s size, and that more aggressive pets may attack Pepper. Furthermore, there were concerns about people with dementia being unable to recognize and work with Pepper without a care partner around, unable to learn how to use or respond to Pepper, and damaging Pepper should they become aggressive.

#### Cost

While the information on the cost of Pepper was not disclosed to the participants, dozens of participants across all three groups were concerned about the cost and affordability of Pepper, and some wondered whether insurance would cover the cost of Pepper. Their concerns on cost were not specifically discussed in detail regarding buying or renting Pepper by individuals or accessing Pepper at facilities. In addition, one comment stated that people from low-income households may not be able to purchase Pepper. In contrast, high-income households that may already have access to personal care assistants may not need Pepper to assist with care.

#### Trust, acceptance, and privacy

All three participant groups had concerns about trust and acceptance of Pepper from people with dementia and their families. One of the most frequently cited concerns was that people with dementia may be frightened and confused by Pepper, and it may be difficult to predict other responses from the people with dementia. To move forward the use of Pepper, it needs to be accepted by the person with dementia and their families. Moreover, some people mentioned that the emotional state, cognitive status, and background of the person with dementia, such as education, culture, and economic status, may influence their acceptance of Pepper. Participants suggested Pepper should be introduced to the individual with dementia early in the disease trajectory to improve acceptance. Notably, one participant mentioned that people might feel embarrassed when other people see them using Pepper outside, resulting in low acceptance. Additionally, some participants in the nonhealthcare workers and care partners groups expressed concerns about privacy issues on personal information.

#### Concerns about pepper replacing human

The concern of Pepper replacing humans was expressed across the three participant groups. They stated that Pepper can assist care but should not replace care partners or healthcare workers since it undermines the human aspect of care and that Pepper should be placed under a care partner's control. Furthermore, one person stated that it was “against his/her religious beliefs to replace humans with robots.”

## Discussion

Pepper has significant potential to engage and support people with dementia. Additionally, Pepper assisted the implementation of COVID-19-related measures and provided support for users during the pandemic (Musa, 2020; Tyagi, 2021). As the development of Pepper improves, it can also support future periods of isolation and promoting engagement among users, including people with dementia. The findings of this study help elucidate the perspectives of nonhealthcare workers, care partners, and healthcare workers in using Pepper to support dementia care. Nonhealthcare workers, care partners, and healthcare workers expressed support towards using Pepper in dementia care and considered it as innovative and useful to support caregiving. Most participants’ comments centered on Pepper's design and functionality and they indicated that Pepper can potentially relieve care partners’ stress. Functions, including assisting with ADLs, promoting independence, facilitating communication, supporting social connections, and providing monitoring, reminding, and companionship, were perceived as helpful in assisting caregiver tasks and benefiting the person with dementia.

Although participants expressed positive perceptions of the use of Pepper in dementia care, some expressed concerns about its appearance and voice. Pepper's appearance was often seen as frightening and unnerving, and the robot's voice was machine-like and difficult to understand. Therefore, when designing Pepper, its appearance and voice should be more natural to improve accessibility and usefulness. Additionally, concerns related to Pepper's ability to perform certain tasks and the robot making mistakes were raised along with the beneficial functions. Thus, Pepper's functionality in performing tasks that the users needed and the potential risk of making mistakes should be addressed prior to implementation in the real world.

One study that explored 52 care partners’ and healthcare workers’ expectations of using Pepper in dementia care had similar results to this study regarding Pepper's functions mentioned above and concerns of Pepper use (Zuschnegg et al., 2021). Results in Zuschnegg et al. (2021), and this study pointed out concerns of Pepper's strength and capability to prevent risky situations, Pepper's inability to respond quickly in emergencies, Pepper causing falls, maintenance problems, technical issues, and the risk of robot abuse in institutions. While participants in the study by Zuschnegg et al. (2021) believed that Pepper could identify danger and notify relevant persons, participants in this study questioned the robot's capacity to accomplish those tasks. This difference could be partly because this study had a larger sample size with broader perspectives that included nonhealthcare workers. Cultural differences may also have influenced the participants’ perspectives regarding robot use (Woods et al., 2021). One review from Woods et al. (2021) pointed out that cultural bias from certain regions may limit the acceptance of social robot use among older adults, and the development of social robots should consider these factors prior to implementation. The study from Zuschnegg et al. (2021) was conducted in Austria, whereas this study's participants were mostly from the United States, which may explain the different results. Moreover, the authors of this study raised concerns about Pepper's ability to identify danger and notify people in the nonhealthcare worker and care partner groups. Pepper's functionality in identifying and responding to dangerous situations needs to be verified in future studies.

The results from this study also added specific suggestions on Pepper's design, functions, and implementation, which can assist with robot development and programming to resolve the function concerns mentioned above. Testing Pepper prior to implementation and using it in a controlled environment with supervision provided, can reduce the risk of Pepper malfunctioning and potentially harming users. The environment in each individual's house can vary widely. Barriers such as stairs, carpets, and tight spaces can impact Pepper's mobility, limiting its use and ability to perform certain tasks. The environment should have even floors and larger spaces to improve Pepper's environmental navigation. Otherwise, the robots should be modified to be capable of working in environments with stairs or tight spaces. Therefore, healthcare facilities can be considered the care setting for pilot testing prior to implementation in individuals’ homes. Adding remote controls and providing technical support can also facilitate the use of Pepper, especially for community-dwelling populations. In addition, the level of cognitive impairment should be considered when implementing Pepper in their care. Those with early-stage dementia may be able to work with Pepper when introduced gradually. However, those with moderate to severe stages of dementia have decreased ability to communicate with others and adjust to new people and environments (Alzheimer's Association, n.d.), leading to challenges in introducing and implementing Pepper in their daily care. Future studies can investigate alternative approaches to program or implement Pepper in people with more severe stages of dementia.

The findings from this study were also similar to another study (Casey et al., 2020) that explored the perceptions of 107 people with dementia, their care partners, and healthcare workers on a machine-like robot, Mario, in providing care to people with dementia. The study was conducted with participants in United Kingdom, Italy, and Ireland. Mario is 1.5 m tall and has a head with two large, animated eyes, a touchscreen on the chest, wheels for mobility, and can be activated by voice or touchscreen (Casey et al., 2020). Pepper has two arms, fingers, and detailed facial expressions, which are lacking in Mario. The results of the study from Casey et al. (2020) are similar to this study in positive perceptions of the robot, improving speech recognition, and adding monitoring and assessment devices in the robot (Casey et al., 2020), which were not reported in Zuschnegg et al. (2021). The study by Casey et al. (2020) was conducted in Europe. The perceptions from Casey et al. (2020) of using Mario in dementia care are similar to this study conducted in the United States. While concerns of using Mario in dementia care in the study from Casey et al. (2020) were similar to this study, participants in the two studies had different concerns about implementing robots. Casey et al. (2020) reported that the loud background noise of the environment frequently impacted Mario's ability to receive and process the user's voice. This was not mentioned in this study because Casey et al. (2020) brought Mario to the participants in person at real-life care settings (e.g., homes, hospital, and long-term care). Participants in this study watched videos about Pepper virtually. Therefore, the real-life environmental background status (e.g., noises) should be considered when implementing Pepper.

Several ethical issues related to Pepper use were mentioned in this study, including cost, privacy, and concerns of the Pepper replacing care provided by humans. Compared to pet-like SARs or SARs with unfamiliar appearance, humanoid robots can provide more detailed facial expressions and body language and better social interaction (Pandey & Gelin, 2018; Prakash & Rogers, 2015). However, the cost could be an issue for users, especially among families with a lower socioeconomic status (SES) that do not have sufficient resources to provide quality care to persons with dementia. A prior quantitative descriptive study examined the ethical issues of using pet-like robots in older adults with and without dementia (Bradwell et al., 2020). Participants were younger adults, and pet-like robots were provided before the survey was distributed (Bradwell et al., 2020). The results showed the most frequently perceived ethical issue was equal access to robots based on an individual's SES (Bradwell et al., 2020), which aligns with the results of this study on the concerns about cost. This could be a critical barrier in implementing any robot into dementia care. Future research may explore the cost and cost-effectiveness of Pepper in dementia care and explore the possibility of insurance coverage or alternative payment options of using Pepper. Also, future research may explore other more affordable technology devices as alternatives.

In terms of safety and privacy issues, Pepper may gather a large amount of personal information from users; thus, protecting personal information is crucial. Zuschnegg et al. (2021) discussed privacy issues during intimate care, and participants felt that Pepper should not participate in these activities (dressing, toileting, using the bathroom, and personal space). Another mixed methods study (Wu et al., 2014) investigated the acceptance of using a machine-like robot among older adults and showed concerns that privacy, including user information and personal space, may be invaded with a robot around. In contrast, the least frequently perceived ethical issue was potential injury and privacy in Bradwell et al. (2020), which differed from the results of this study. The difference in findings can be partly because Bradwell et al. (2020) used a pet-like robot, which did not collect as much user information or provide as much assistance in care activities as humanoid robots. This study also included care partners’ and healthcare workers’ perspectives, which may have different privacy concerns compared to participants who had limited caregiving experiences in the study from Bradwell et al. (2020). When designing Pepper, privacy and personal information protection should be considered. Future research may further explore approaches to only collecting essential information necessary for the care and ensuring data storage safety.

Participants in this study had opposing views about whether Pepper should be human-like. This issue was also reported in another study (Casey et al., 2020). Participants in Casey et al. (2020) mentioned that Mario could be more useful for social interaction with more human-like characteristics, such as facial recognition and more autonomy. The issue of the Pepper being more or less human-like was mixed in this study. These different results are explained by using different types of robots used in the two studies. Casey et al. (2020) used a machine-like robot, and this study used a humanoid robot. One benefit of more human-like robots is that they may facilitate social interactions due to more detailed human-like facial expressions and gestures. However, participants had concerns about Pepper replacing humans when they look too human-like. It is possible that people with more severe dementia may believe that Pepper are humans, posing an ethical issue related to deceiving the person with dementia.

Bradwell et al. (2020) also reported that around 30% of the participants were concerned about the ethical issues of companion robots in reducing human contact in older adults with and without dementia. Participants in another study had similar concerns and stated that people with dementia need human interactions and that Pepper cannot provide the same care that humans can provide (Casey et al., 2020). This suggests that Pepper may be a resource to assist and supplement caregiving efforts but should not replace human care partners or healthcare workers (Zuschnegg et al., 2021). Therefore, more research is needed to explore the meaning of humanoid robots, the degree of services Pepper should ideally provide, and how Pepper could enhance the care provided by care partners and healthcare workers, and to examine the differences in needs for people at different stages of dementia.

Several themes were identified across the categories of views and perceptions. First, Pepper should be personalized to each individual's background, preferences, needs, and cognitive and functional level, including its appearance, language, communication, and function. This is in line with the person-centered care approach proposed by Kitwood (1997), which states that comfort (trust), attachment (familiarity), inclusion (being involved), occupation, and identity were needs that should be satisfied in people with dementia psychologically. Therefore, this theory can be considered when programming Pepper, such as naming Pepper as someone familiar or starting conversations according to the person's background with dementia. Physical needs should be considered as well. For example, Pepper should have lip movements or subtitles for people with hearing impairment.

Second, Pepper's size, structure, sound, and appearance may impact its use in dementia care. There were negative comments about the sound and appearance of Pepper. Its appearance and voice are perceived as irritating and frightening, impacting user acceptance. The child-like size and wheel structure rather than legs may limit its ability to bear weight, move around uneven floors, and respond to emergencies quickly. Therefore, Pepper's mobility design needs to be improved for better implementation in individuals’ homes. Pepper's appearance and voice should be less irritating, frightening, and machine-like quality for better acceptance.

Third, there were concerns about the acceptance, trust, and use of Pepper among people with dementia and their families. People with dementia may be confused, frightened, or unable to recognize Pepper, leading to their families feeling uncomfortable leaving the person with dementia alone with Pepper. Dignity and embarrassment may be an issue in certain individuals with dementia when being seen with Pepper. The cognitive function and preferences of the person with dementia can also impact their acceptance and trust towards Pepper. To improve acceptance, some participants suggested introducing Pepper early in the course of dementia progression and gradually over time. This could help people with dementia, their care partners, and healthcare workers become familiar with Pepper and thus increase acceptance and trust. Incorporating their cognitive level and preferences can also facilitate a successful implementation of Pepper in dementia care.

## Strengths and Limitations

This study has a relatively large sample size (*n*  =  194) and diverse geographic characteristics, primarily within the United States. However, several limitations of this study have been identified. The qualitative responses from participants were through one open-ended question from an online survey and no follow-up questions were asked. This limits the ability for researchers to clarify further or acquire more details on responses. The sample size of healthcare workers was small (*n*  =  11). Therefore, the understanding of healthcare workers’ perspectives on using Pepper is limited. People with dementia were not included in this study, which limits the understanding of how Pepper can be helpful for people with dementia directly from their perspective. Moreover, this study specifically focused on Pepper and its use in persons with dementia, so the results of this study cannot be generalizable to other types of robots or other populations. However, despite the limitations, this study provided insights of improving specific designs, functions, and logistics on Pepper, which can inform future robot designs to fit the needs of users and persons with dementia**.**

## Implications for Practice and Research

This study explored the perceptions of Pepper use in people with dementia from the perspectives of nonhealthcare workers, care partners, and healthcare workers. The findings provide guidance on the development of Pepper and their clinical implications in future research. Overall, the findings suggest that the public, care partners, and healthcare workers support using Pepper for people with dementia and their care partners and healthcare workers. The results identified key areas where Pepper could be helpful for people with dementia and their care providers. While the results provide a good place to start, much work remains to be done. Future research may examine the use of Pepper in those specific areas and evaluate its effect on people with dementia and their care partners and healthcare workers. Additionally, the results highlighted the need for improvement in the Pepper's design, function, and logistic issues, which need to be addressed in future research. The implementation of Pepper should also consider proper robot use, environmental navigation, and the safety of people with dementia. The protocol to personalize Pepper to meet individuals’ needs, background, functions, cognitive level, and preference needs to be developed and evaluated as well. Future research should further explore the perceptions of Pepper use with more in-depth interviews to collect more detailed information. It is also important to understand the perceptions of people with dementia.

## Conclusion

Cognitive decline negatively affects the independence of individuals with dementia, their QOL, need for caregiving, and care partner stress. The findings suggest that nonhealthcare workers, care partners, and healthcare workers had positive perceptions about using a Pepper robot in dementia care with some concerns and recommendations for improvement. Participants perceived that Pepper has the potential to assist with ADLs, promote engagement in physical and social activity for people with dementia, and support caregiving tasks. The findings suggest that there is room for improvement in Pepper's design and function, including its environmental navigation capacity and cost. Finally, individual preferences and needs of the user should be considered when implementing Pepper in dementia care.
